# Study on cerebrospinal fluid meropenem, vancomycin and tigecycline monitoring in patients with central nervous system infection following neurosurgery under different drug regimens

**DOI:** 10.3389/fphar.2025.1666168

**Published:** 2025-09-30

**Authors:** Xiaoman Zhao, Yanan Qiao, Qing Xie, Zheng Zhang, Yan Song, Jianbang Kang, Jinchuan Li, Jinju Duan

**Affiliations:** ^1^ School of Pharmacy, Shanxi Medical University, Taiyuan, China; ^2^ Department of Pharmacy, Second Hospital of Shanxi Medical University, Taiyuan, China; ^3^ Department of Pharmacy, Shanxi Eye Hospital, Taiyuan, China; ^4^ Department of Neurosurgery, Second Hospital of Shanxi Medical University, Taiyuan, China

**Keywords:** cerebrospinal fluid, central nervous system infection, neurosurgery, antibiotic monitoring, different drug regimen

## Abstract

**Introduction:**

Central nervous system infection (CNSI) following neurosurgery is challenging to treat and carries a high risk of recurrence, morbidity, and mortality. Low CNS penetration of antibiotics may contribute to poor clinical outcomes from CNS infections. Different drug regimens also suggested variable impacts on clinical outcomes. This study aims to measure the cerebrospinal fluid (CSF) concentration of meropenem, vancomycin and tigecycline in patients with CNSI following neurosurgery and thus evaluate the differential therapeutic efficacy of different drug regimens.

**Methods:**

Patients who received meropenem, vancomycin and/or tigecycline for highly suspected or confirmed bacterial CNSI following neurosurgery were recruited from a tertiary hospital in Shanxi from January 2021 through December 2022. The concentrations of these three antibiotics in CSF and/or plasma were determined by high-performance liquid chromatography (HPLC) or enzyme immunoassay. Relevant pharmacokinetic/pharmacodynamic (PK/PD) parameters were assessed using DAS 2.0 software. Body temperature, biochemical examination and bacterial culture results were collected to evaluate efficacy.

**Results:**

In total, 55 CSF and ten plasma samples obtained from ten patients were included in this study. In particular, of five patients who had a positive CSF culture, four achieved culture conversion to negative. Nine individuals successfully achieved CSF, blood tests, or body temperature improvement. Only one patient showed no improvement at discharge.

**Conclusion:**

The CSF concentration and PK/PD parameters of meropenem, vancomycin, and tigecycline in patients with CNSI following neurosurgery featured large inter-individual variation. Different drug regimens can partially improved the outcomes of such patients, but monitoring of potential adverse reactions is required.

## 1 Introduction

Central nervous system infection (CNSI) following neurosurgery is a serious complication that requires immediate recognition and treatment. These infections are associated with a high mortality rate and poor prognosis if not treated early. Appropriate antibacterial drugs should be given promptly when bacterial CNSI occurs (Kulikov et al., 2022; Zhao et al., 2022; Gatos et al., 2023). Meropenem and vancomycin, with antimicrobial activity against the majority of Gram-negative and Gram-positive bacteria, are recommended for the empirical treatment of these challenging infections (Ye et al., 2022; Chen et al., 2023; Blassmann et al., 2019). Tigecycline has been used for the treatment of infections caused by Enterobacteriaceae that demonstrate carbapenem resistance (Teo et al., 2023; Yaghoubi et al., 2022).

Unfortunately, these antibiotics offering great promises for the treatment of CNSI have limited brain uptake due to the blood-brain barrier (BBB) (Naseri Kouzehgarani et al., 2021). Selecting an antimicrobial regimen penetrating the BBB and maintaining sufficient concentrations is severely challenged (König et al., 2023; Rezzonico et al., 2024). Different neurosurgical procedures resulted in varying degrees of BBB disruption followed by different abilities of drug molecules to penetrate the BBB. Moreover, BBB showed different extents of recovery over a different postoperative period. Thirdly, the treatment regimen had a significant impact on the antibiotic concentration in CSF. Fourthly, the management of bacterial CNSI was complicated by difficulties in obtaining samples for microbiological analysis. While the pharmacokinetic profile in serum could be easily monitored, the drug concentration in brain tissue was more difficult to assess. Most drugs did not readily equilibrate between serum and brain, which was the result of the sheltering property of the BBB with its tight junctions and transporter systems (Fong et al., 2024). The free serum concentration often fails to be a reasonable surrogate for brain exposure (Naseri Kouzehgarani et al., 2021). Lastly, there was likely significant inter- and intra-individual variation in blood and CSF antimicrobial concentration, with potential clinical covariates including comorbidities, fluid balance, and albumin status, to name a few (Arkell et al., 2022; Wicha et al., 2024).

All of these situations further highlight the necessity for CSF antibiotic monitoring in patients with CNSI following neurosurgery to achieve clinical efficacy and prevent toxicity. Clinical data from this type of patient on meropenem, vancomycin and tigecycline concentrations in the CSF are limited. In the present study, 65 samples (55 CSF and ten plasma) obtained from ten patients treated with different regimens of antibiotics were included. Meropenem, vancomycin and tigecycline levels in CSF and/or plasma were measured and the pharmacokinetic characteristics were analyzed. Therefore, we hope that the results we have described will represent a useful reference for assisting practicing clinicians in the management of CNSIs following neurosurgery.

## 2 Materials and methods

### 2.1 Study design

This study was conducted in the Department of Neurosurgery at a tertiary hospital in Shanxi from January 2021 through December 2022. Our study was approved by the ethics committee of our hospital (No. 2019YX-278) and had been granted a waiver of informed consent. The inclusion criteria were as follows: highly suspected or confirmed bacterial CNSI post neurosurgery, use of meropenem, vancomycin and/or tigecycline, and at least one monitoring of the study drug. The exclusion criteria were as follows: a history of allergy to any of the trial’s drugs, a duration of medication use less than 48 h, severe liver and kidney dysfunction, and patients who did not agree to participate in the study. Demographic, clinical and laboratory data were collected. To protect patient confidentiality and private health information, identifying information was not included in this manuscript.

The diagnosis of CNSI was as follows: 1 CSF cultures are positive. 2 A temperature of more than 38 °C, headache, neck stiffness, meningeal irritation signs and cough and/or sore throat in the absence of another known cause, increased intracranial pressure or irritability. 3 Decreased CSF glucose, elevated CSF protein level, and/or increased CSF white blood cell (WBC) counts. 4 WBC count of more than 10.0 × 10^9^/L, neutrophil percentage of greater than 80%. Patients who met diagnostic criteria (1) and either of diagnostic criteria (2), (3) or (4) were diagnosed with confirmed bacterial CNSI. Patients who met any two diagnostic criteria (2), (3) or (4) were diagnosed with highly suspected CNSI.

Penetration of drugs into CSF was described using the C_CSF_/C_plasma_, which was calculated by dividing the cerebrospinal fluid drug concentration by the plasma drug concentration. To reflect the integrity of the BBB, the age-dependent albumin quotient (QAlb) was assessed (Castellazzi et al., 2020).

### 2.2 Evaluation of the therapeutic effect

The response evaluation of our study was as follows (Wang et al., 2024): 1 A negative CSF bacterial culture. 2 Significant improvement in routine CSF examination, including higher CSF glucose levels, lower CSF protein levels, and CSF WBC (white blood cell) counts. 3 Significant improvement in routine blood tests (lower WBC counts, absolute neutrophil count and percentage of neutrophils). 4 Significant improvement in inflammatory markers (lower CRP or PCT 5 Significant improvement in the clinical presentation of CNSI and body temperature. The effective treatment was identified if patients fulfilled more than two response criteria. Otherwise, it was recorded as an ineffective response.

### 2.3 Sample collection for drug level determination

CSF and plasma samples were collected at certain practically one or more time points (0.5, 1, 1.5, 2, 3, 4, 5, 6, 7, 8, 10, and/or 12 h) after the start of the antibiotic administration trials. Lumbar cisternal drainage was used for meropenem, vancomycin, and tigecycline concentration testing and routine CSF examination. Lumbar puncture was used for routine CSF examination, a procedure that demands specific professional knowledge from the operator.

The concentrations of meropenem were determined using a high-performance liquid chromatography (HPLC) assay (Berska et al., 2024; Smith, 2012; Milla et al., 2020). Both CSF and plasma samples were extracted through the methanol protein precipitation method. The analysis of meropenem levels was conducted on the Alliance e2695 liquid chromatograph (Waters, United States), equipped with a Waters 2998 photo-diode array detector at 30 °C and a flow rate of 1 mL/min. The detection wavelength was set to 298 nm. Mobile phase A was methanol. Mobile phase B was phosphate-buffered saline containing 0.005 mol/L KH_2_PO_4_ and 0.005 mol/L Na_2_HPO_4_. They were eluted with a gradient of 10%–20% A in 10 min, 20% A for 2 min, 20%–10% A in 1 min, and 10% A for 2 min. Meropenem reference substance (Lot No. 130506-202004) and Ceftazidime (the internal standards, Lot No. 130484-201806) were purchased from the National Institutes for Food and Drug Control of China. The standard curves of meropenem in CSF and plasma ranged from 0.25 to 20 µg/mL and 1.0 to 80 µg/mL, respectively. Both correlation coefficients of the standard curves were 0.9998.

Vancomycin levels in CSF and plasma were determined using enzyme immunoassay based on the instructions on the corresponding assay kit (Siemens, United States) (Chu et al., 2020). CSF and plasma samples were centrifuged at 4,000 r/min for 5 min before a fully automatic biochemistry analyzer (VIVA-E, Siemens, United States). The standard curves of vancomycin ranged from 0 to 50 µg/mL.

CSF for tigecycline testing was pretreated as described below (Huang et al., 2022). Firstly, 600 μL of releasing agent (ACP-1, Hunan Demite Instruments Ltd., China) was added into an EP tube, and 200 μL of CSF sample was then added. The supernatant was collected after high-speed centrifugation (14,000 r/min for 8 min). A protectant (60 μL ACG, Hunan Demite Instruments Ltd., China) was added and mixed before HPLC analysis. Two-dimensional LC (FLC 2420, Hunan Demite Instruments Ltd.) was performed using a phenyl column (Aston SNX5, 50 mm * 4.6 mm, 5 μm) connected to another column (Aston SCB, 250 mm * 4.6 mm, 5 μm). The flow rate of pump A was set at 1.20 mL/min, pump B at 0.01 mL/min, and pump C at 1.0 mL/min. The column was held at 40 °C, and the injection volume was 200 μL. The standard curves of tigecycline in CSF ranged from 49.305 to 2536.24 ng/mL. The correlation coefficient of the standard curve was 0.9998. Guidelines of the FDA for Bioanalytical Method Validation (FDA, 2018) were followed for above method validation.

### 2.4 Statistical analysis

Qualitative variables were presented as frequencies (%) and mean ± standard deviation (SD) for continuous variables. The concentration-time curve of the drug in CSF was analyzed using DAS 2.0 based on a two-compartment model. Individual pharmacokinetic parameters were calculated using non-compartmental analysis (NCA).

## 3 Results

### 3.1 Characteristics of the patients

Ten patients met the inclusion criteria for enrollment in the study (Table 1). Half were male and half female. The mean (SD) age was 58.3 years (9.7), BMI 23.2 kg/m^2^ (3.2), and hospital stay 36.7 days (11.8). One patient (10.0%) had normal albuminuria, six (60.0%) had normal creatinine, five (50%) had normal liver function (AST and ALT), and other renal and liver function parameters are presented in Supplementary Table S1.

**TABLE 1:** Clinical characteristics of the study population (N = 10)^a^.

Patient ID	Gender	Age, years	BMI, kg/m^2^	Hospital stay, days	Albumin, g/L	Creatinine, µmol/L	ALT, U/L	AST, U/L
Ⅰ	F	65	24.4	33	27.9	45	23.6	47.2
Ⅱ	M	67	21.9	19	30.7	69	14.1	23.1
Ⅲ	M	47	17.7	28	38.8	124	8.0	18.3
Ⅳ	F	71	25.4	29	41.5	57	22.9	23.1
Ⅴ	F	43	22.3	42	29.5	32	24.3	59.4
Ⅵ	F	62	26.7	55	36.5	39	17.6	30.4
Ⅶ	M	54	18.7	27	31.2	60	14.1	21.8
Ⅷ	F	56	22.5	35	33.3	33	41.9	37.0
Ⅸ	M	50	25.7	22	32.4	47	36.2	35.9
Ⅹ	M	68	26.7	49	31.2	49	112.4	93.3
Mean ± SD	NA^b^	58.3 ± 9.7	23.2 ± 3.2	36.7 ± 11.8	33.3 ± 4.3	55.5 ± 26.8	31.5 ± 30.2	39.0 ± 23.0

^a^ BMI, body mass index; ALT, alanine transaminase; AST, aspartate aminotransferase; Mean ± SD, standard deviation.

^b^ NA, not available; M, male; F, female.

Diagnosis, antibiotic regimen, and other related information can be found in Table 2. The mean (SD) time to post-operative infection was 9.7 days. Of these patients, two were given meropenem only (Patients Ⅰ and Ⅱ), two received vancomycin only (Patients Ⅲ and Ⅳ), and one was treated with tigecycline only (Patient Ⅴ). Four patients received both meropenem and vancomycin therapy (Patients Ⅵ, Ⅶ, Ⅷ, and Ⅸ). There was also a patient receiving both meropenem and tigecycline (Patient Ⅹ). The CSF and plasma samples collected were 55 and 10, respectively. For Patients Ⅰ, Ⅱ, Ⅴ, and Ⅹ, CSF samples were taken at different time points during a dosing interval after steady-state reached. For the remaining six patients, CSF samples were taken at indicated time points after administration. Three received both CSF and plasma monitoring at least once (Patients Ⅵ, Ⅷ, and Ⅸ).

**TABLE 2:** Diagnosis, surgery, sample collection, and antibiotic regimen of the study population^a^.

Patient ID	Diagnosis	Neurosurgery	Time to post-operative infection, days	CSF cultures^b^	Antibiotic regimen^c^	CSF		Plasma sample number
						Sample number	Sampling route^d^	
Ⅰ	1. Subdural hematoma	1. Durotomy with the evacuation of Subdural hematoma	8	NA	MEM A	5	LD	0
	2. Cerebral hernia	2. Decompressive hemicraniectomy						
Ⅱ	1.Intraventricular hemorrhage	Evacuation of intracerebral hematoma	3	NA	MEM A	6	LD	0
	2. Cerebral hernia							
Ⅲ	1.Hypertensive intracerebral hemorrhage	External ventricular puncture drainage	11	*Sep*	VAN A	1	LD	0
	2. Secondary Intraventricular hemorrhage							
Ⅳ	Meningioma	Brain lesion resection	11	NA	VAN A	1	LD	0
Ⅴ	1. Subarachnoid hemorrhage	Burr-hole drainage	15	*STM; Sep; Efa*	TGC	8	LD	0
	2. Hydrocephalus							
	3. Intraventricular hemorrhage							
	4. Cerebral hernia							
Ⅵ	1. Hemorrhage from the basal ganglia into the ventricle	Placement of external ventricular drainage device	14	*P. larvae*	MEM B	2	LD	1
	2. Hydrocephalus				VAN B	1		1
Ⅶ	1. Encephalopyosis	Frontal lesion resection	4	NA	MEM B	1	LD	0
	2. Meningitis				VAN A	1		0
Ⅷ	1.Intracranial aneurysm	Resection of intracranial vascular malformations	5	NA	MEM A	4	LD	1
	2. Subarachnoid hemorrhage				VAN A	3		1
Ⅸ	Intracranial aneurysm	Cerebral Aneurysm clipping	8	*B. altitudinis*	MEM B Followed by MEM C	4	LD	3
					VAN B	3		3
Ⅹ	1. Traumatic brain injury	1. Durotomy with the evacuation of Subdural hematoma	18	*A. baumannii*	MEM B	8	LD	0
	2. Hydrocephalus	2. Decompressive hemicraniectomy			TGC	7		0

^a^ CSF, cerebrospinal fluid.

^b^ NA, the CSF, culture was not available; *Sep*, *Staphylococcus* epidermidis; *STM*, stenotrophomonas maltophilia; *Efa*, *Enterococcus* faecium; *P. larvae*, Paenibacillus larvae; *B. altitudinis, Bacillus* altitudinis; *A. baumannii*, *Acinetobacter* baumannii.

^c^ Treatments were administered by intravenous infusion unless specified otherwise. MEM A, meropenem, 1 g q8h; MEM B, meropenem, 2 g q8h; MEM C, meropenem, 2 g, continuous minipump infusion over 3 h every 8 h; VAN A, vancomycin, 1 g q12h; VAN B, combination of intravenous (1 g, q12h) and intrathecal administration (10 mg q24h before intrave-nous delivery from Day 1 to Day 3); TGC, tigecycline 100 mg as a loading dose followed by 50 mg every 12 h.

^d^ LD, lumbar cisternal drainage; EVD, external ventricular drain; LP, lumbar puncture.

**FIGURE 1:**
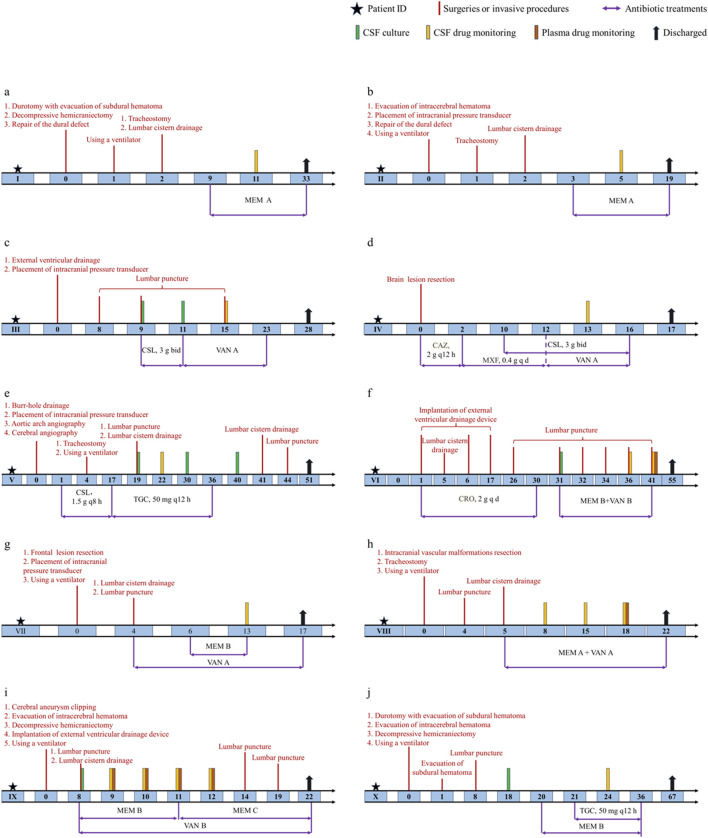
Schematic representation of the treatment process of ten patients. **(a)** Patient Ⅰ. **(b)** Patient Ⅱ. **(c)** Patient Ⅲ. **(d)** Patient Ⅳ. **(e)** Patient Ⅴ. **(f)** Patient Ⅵ. **(g)** Patient Ⅶ. **(h)** Patient Ⅷ. **(i)** Patient Ⅸ. **(j)** Patient Ⅹ. Treatments were administered by intravenous infusion unless specified otherwise. CSL, cefoperazone-sulbactam; CAZ, ceftazidime; MXF, moxifloxacin; CRO, ceftriaxone.

The corresponding schematic representation of the treatment process of these ten patients is shown in Figure 1. We defined day 0 as the time of the first neurosurgery during hospitalization. Patient Ⅰ was treated using the meropenem A protocol (1 g intravenous infusion every 8 h) from days 9 to 33, and meropenem CSF concentrations were monitored at day 11. Patient Ⅰ was discharged on postoperative day 33. Patient Ⅱ was offered the same antibiotic regimen as patient Ⅰ from days 3 to 19. We examined CSF meropenem concentrations from patient Ⅱ on postoperative day 5, and he was discharged on the 19th postoperative day. Patient Ⅲ had a positive CSF culture for *Staphylococcus epidermidis* and was treated with cefoperazone-sulbactam (3 g, two times per day) from days 9 to 11. The antibiotic protocol was amended to the vancomycin A regimen (1 g intravenous infusion every 12 h) from days 11 to 23. The CSF vancomycin level was monitored on postoperative day 15, and the patient was discharged on day 28. For patient Ⅳ, ceftazidime (2 g, every 12 h) was first used. The antibiotic treatment scheme was modified to moxifloxacin (0.4 g, once per day) from days 2 to 12 and then the vancomycin A regimen from 12 to 16. In the meantime, patient Ⅳ was concurrently treated with cefoperazone-sulbactam (3 g, two times per day) from days 10 to 16. The CSF vancomycin level was monitored on postoperative day 13, and the patient was discharged on day 17.

For patient Ⅴ, CSF samples were culture-positive on days 19, 30 and 40. The antibiotic treatment options were cefoperazone-sulbactam (1.5 g, every 8 h) from days 1 to 17 and were switched to tigecycline (100 mg as a loading dose followed by 50 mg every 12 h) for the subsequent 20 days. The CSF vancomycin level was monitored on postoperative day 22, and patient Ⅴ was discharged on day 51. For patient Ⅵ, ceftriaxone (2 g, once per day) was first used in the first 30 days after surgery. CSF samples were culture positive on day 31, and the antibiotic protocol was amended to meropenem B regimen with vancomycin B regimen from days 31 to 41. The plasma drug levels were monitored on postoperative day 41 and CSF concentrations on days 36 and 41. Patient Ⅵ was finally discharged on day 55. Patient Ⅶ was treated using the vancomycin A protocol from days 4 to 17. In the meantime, this patient received the meropenem B regimen from days 6 to 13. The CSF vancomycin and meropenem levels were monitored on postoperative day 13, and the patient was discharged on day 17. Patient Ⅷ received combined therapy (meropenem A plus vancomycin A) from days 5 to 22. The plasma drug levels were monitored on postoperative day 18 and CSF concentrations on days 8, 15 and 18. Patient Ⅷ was discharged on day 22. Patient Ⅸ had positive CSF culture on day 8 and was treated with meropenem plus vancomycin from days 8 to 12. CSF and plasma antibiotic concentrations were measured for four consecutive days. Patient Ⅸ was discharged on day 22. Patient Ⅹ had positive CSF culture on day 18. Scheduling of meropenem B was initiated on day 20. Tigecycline was added on day 21. Meropenem and tigecycline CSF concentrations were monitored on day 24. Antibiotic treatment was stopped on day 36. Patient Ⅹ was finally discharged on day 67.

### 3.2 Meropenem monitoring

CSF and/or plasma meropenem concentrations at different time points are presented in Table 3. Over 6 h, the concentrations of meropenem in CSF ranged between 0.65 and 5.50 μg/mL. Meropenem plasma levels ranged from 0 to 38.1 μg/mL over 1.5 h. Of three patients receiving the meropenem A regimen, trough concentrations of patient Ⅷ measured is 1.08 μg/mL. This patient’s BBB permeability was 34.3%. For those who were treated with the meropenem B regimen, the range of trough concentrations in the CSF was 1.22 to 2.18 μg/mL. For Patient Ⅵ, the BBB permeability was 56.7%. Patient Ⅸ’s meropenem Regimen B was also administered via intravenous infusion. Meropenem levels in CSF and plasma were measured (3.66 and 38.10 μg/mL), and the corresponding BBB permeability at C_1.5 h_ was 9.6%.

**TABLE 3:** Meropenem monitoring results^a^.

Patient ID	Ⅰ	Ⅱ	Ⅵ	Ⅶ	Ⅷ	Ⅸ	Ⅹ
MEM Regimen	A	A	B	B	A	B+C	B
CSF C_trough_, μg/mL	NA	NA	1.22	2.18	1.08	NA	NA
CSF C_0 h_, μg/mL	NA	NA	NA	NA	NA	NA	1.08
CSF C_0.5 h_, μg/mL	1.52	1.03	NA	NA	1.45	2.49^b^	1.54
CSF C_1.0 h_, μg/mL	2.43	1.53	NA	NA	NA	NA	1.61
CSF C_1.5 h_, μg/mL	NA	NA	NA	NA	NA	3.66	NA
CSF C_2.0 h_, μg/mL	3.52	1.63	NA	NA	NA	NA	2.83
CSF C_3.0 h_, μg/mL	2.90	2.01	NA	NA	NA	NA	5.50
CSF C_4.0 h_, μg/mL	1.57	1.65	NA	NA	NA	NA	2.71
CSF C_6.0 h_, μg/mL	NA	1.30	NA	NA	NA	NA	1.79
Plasma C_trough_, μg/mL	NA	NA	2.15	NA	3.15	0	NA
Plasma C_0.5 h_, μg/mL	NA	NA	NA	NA	NA	14.10^b^	NA
Plasma C_1.5 h_, μg/mL	NA	NA	NA	NA	NA	38.10	NA

^a^ C_trough_, concentration at trough; NA, not available.

^b^ Samples were collected under schedule C of meropenem.

**FIGURE 2:**
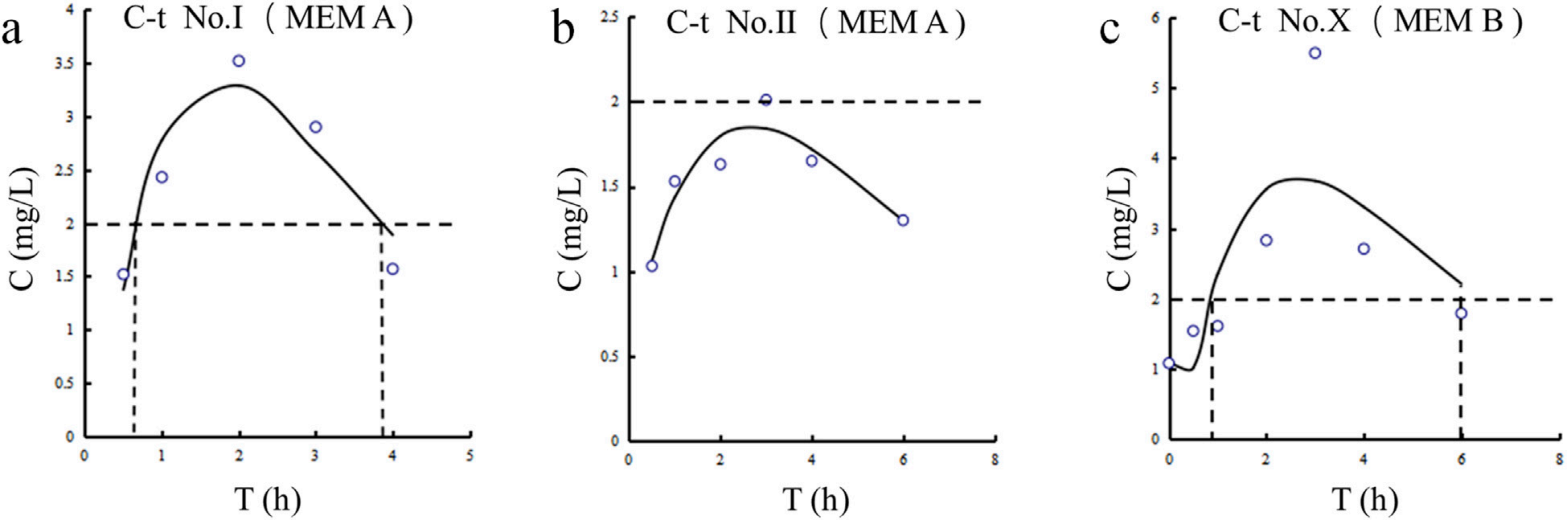
Meropenem concentration-time curves in CSF for Patients Ⅰ, Ⅱ, and Ⅹ. **(a)** Patient Ⅰ. **(b)** Patient Ⅱ. **(c)** Patient Ⅹ.

The CSF concentration-time profiles for patients Ⅰ, Ⅱ and Ⅹ are shown in Figure 2. The corresponding pharmacokinetic parameters are presented in Table 4. The CSF half-lives (T_1/2_) of meropenem in the three patients were 1.717, 4.896, and 1.978 h. The corresponding time to peak concentration was no more than 3 h. The highest peak plasma concentration (C_max_) and area under the concentration-time curve (AUC) were observed in patient Ⅹ.

**TABLE 4:** The pharmacokinetic parameters of meropenem in CSF^a^.

Patient ID	T_1/2_, h	T_max_, h	C_max_, μg/mL	${\text{AUC}}_{0-\infty }$ , h·μg/mL	%T > MIC
Ⅰ	1.717	2	3.52	13.96	40.1
Ⅱ	4.896	3	2.01	18.16	0
Ⅹ	1.978	3	5.50	21.19	64.4
Mean ± SD	2.86 ± 1.76	2.67 ± 0.58	3.68 ± 1.75	17.77 ± 3.63	34.83 ± 32.52

^a^ T_1/2_, half-life; Tmax, time to achieve the maximum observed concentration; Cmax, maximum concentration; AUC, area under the concentration-time curve; MIC, minimum inhibitory concentration; %T > MIC% = (Time above MIC/Dosing Interval) × 100%, the percentage of time that drug concentrations are higher than the MIC. Given that the current breakpoint for common Gram-negative bacteria causing a CNS, infections is 2 μg/mL (Alshaer et al., 2022).

### 3.3 Vancomycin monitoring

CSF and plasma vancomycin concentrations are presented in Table 5. For patients treated with the vancomycin A regimen, the mean trough concentrations in CSF were 2.22 ± 0.87 μg/mL, ranging from 0.7 to 3.3 μg/mL. For two patients treated with the vancomycin B regimen, CSF concentrations at trough were 4.7 μg/mL in patient Ⅵ. Concentrations measured in patient Ⅸ was 0.8 μg/mL. For patient Ⅷ, the BBB permeability was 39.8%. The overall proportion of individuals who improved after antibacterial therapy was 90%.

**TABLE 5:** Vancomycin monitoring results^a^.

Patient ID	Ⅲ	Ⅳ	Ⅵ	Ⅶ	Ⅷ	Ⅸ
VAN Regimen	A	A	B	A	A	B
CSF C_trough_, μg/mL	0.7	1.9	4.7	2.4	3.3	0.8
CSF C_2.0 h_, μg/mL	NA	NA	NA	NA	NA	7.8
Plasma C_trough_, μg/mL	NA	NA	7	NA	8.3	2.2
Plasma C_2.0 h_, μg/mL	NA	NA	NA	NA	NA	10.3

^a^ NA, not available.

### 3.4 Tigecycline monitoring

Patients receiving tigecycline 100 mg as a loading dose followed by 50 mg every 12 h. After intravenous drip, samples from patients Ⅴ and Ⅹ were collected on Day 6 and Day 4, respectively. Tigecycline CSF levels are presented in Table 6.

**TABLE 6:** Tigecycline monitoring results^a^.

Patient ID	Ⅴ	Ⅹ
CSF C_trough_, ng/mL	NA	NA
CSF C_1.0 h_, ng/mL	17.367	35.979
CSF C_1.5 h_, ng/mL	21.199	37.353
CSF C_2.0 h_, ng/mL	30.605	42.614
CSF C_2.5 h_, ng/mL	32.294	NA
CSF C_3.0 h_, ng/mL	34.307	55.214
CSF C_4.0 h_, ng/mL	26.007	77.682
CSF C_5.0h_, ng/mL	NA	63.276
CSF C_6.0 h_, ng/mL	20.313	43.053
CSF C_7.0h_, ng/mL	NA	39.412
CSF C_8.0h_, ng/mL	21.545	NA

^a^ NA, not available.

**FIGURE 3:**
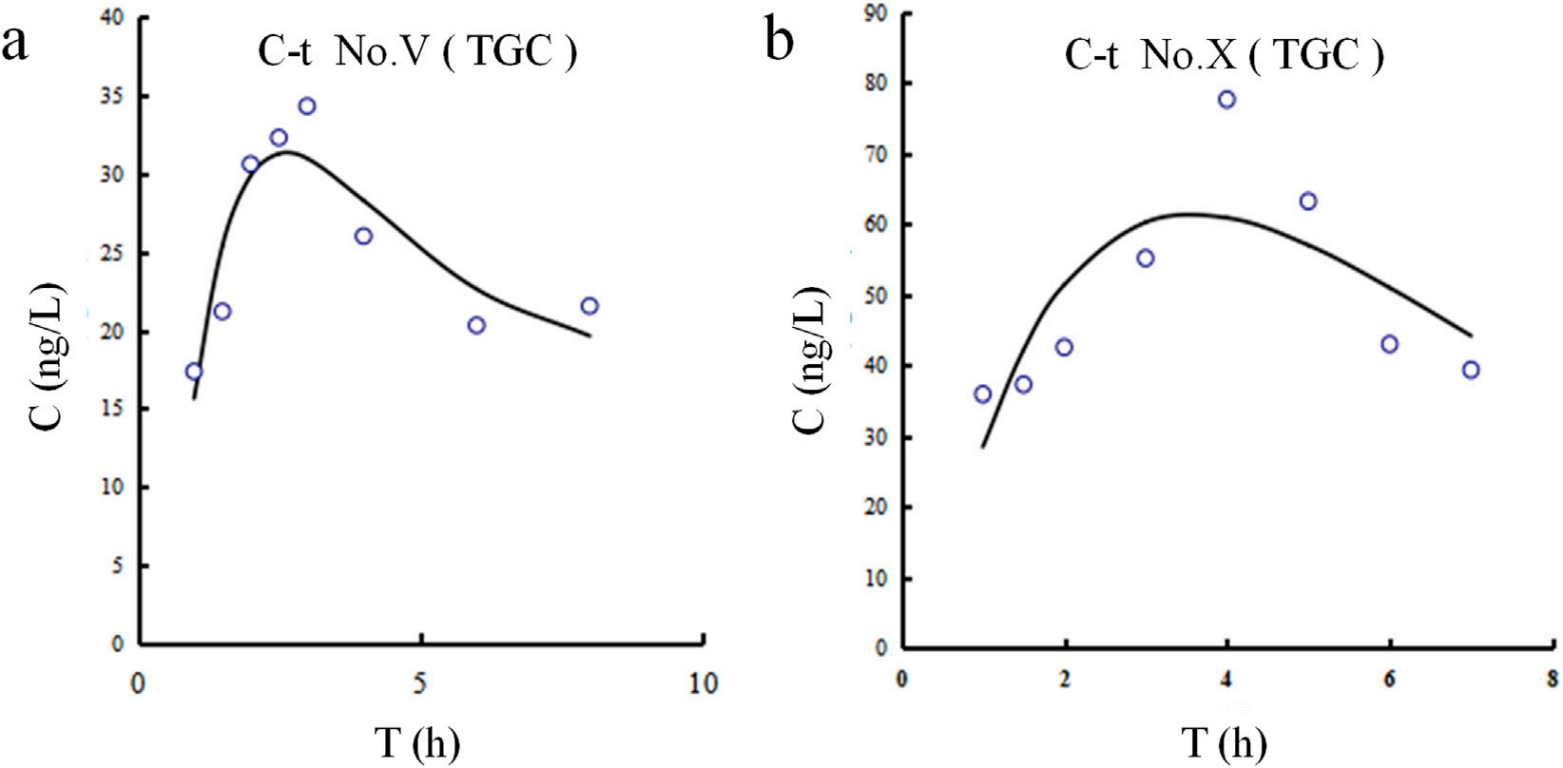
Tigecycline concentration-time curves in CSF for Patients Ⅴ and Ⅹ. **(a)** Patient Ⅴ. **(b)** Patient Ⅹ.

The CSF concentration-time profiles for both patients are shown in Figure 3. The corresponding pharmacokinetic parameters are displayed in Table 7. The half-lives of tigecycline in the CSF of the two patients were 5.251 and 8.253 h, respectively, and the AUC was 295.39 and 806.78 h.ng/mL, respectively. There were large differences between patient Ⅴ and Ⅹ on these measures’ parameters.

**TABLE 7:** The pharmacokinetic parameters of tigecycline in CSF.

Patient ID	T_1/2_, h	T_max_, h	C_max_, ng/mL	${\text{AUC}}_{0-\infty }$ , h·ng/mL
Ⅴ	5.251	3	34.307	295.39
Ⅹ	8.253	4	77.682	806.78
Mean ± SD	6.75 ± 2.12	3.50 ± 0.71	55.99 ± 30.67	551.09 ± 361.61

### 3.5 Therapeutic effect

In order to analyze the effectiveness of the antibacterial treatment, infection-related information is measured before and after treatment (Table 8). CSF culture was mainly taken to diagnose CSF infections. In particular, of five patients with a positive CSF culture, four (Patients Ⅲ, Ⅴ, Ⅸ, and Ⅹ) achieved culture conversion to negative. The other patient (Patient Ⅵ) experienced an improvement in CSF WBC count, neutrophil percentage, and body temperature. Four of the remaining five individuals (Patients Ⅱ, Ⅳ, Ⅶ, and Ⅷ) successfully achieved CSF, blood tests, or body temperature improvement/almost improvement. Only one patient (Patient Ⅰ) showed no improvement at discharge. The overall proportion of individuals who improved after antibacterial therapy was 90%.

**TABLE 8:** The efficacy evaluation of antimicrobial agents in ten patients^a^.

Patient ID	CSF culture		CSF glucose, mmol/L		CSF protein, g/L		CSF WBC count, × 10^6^/L		CSF RBC count, × 10^6^/L		Blood WBC count, × 10^9^/L		NEU#, × 10^9^/L		NEU%, %		CRP, mg/L		PCT, ng/mL		Body temperature, °C		Clinical outcome
Before or after antibiotic treatment^b^	Before	After	Before	After	Before	After	Before	After	Before	After	Before	After	Before	After	Before	After	Before	After	Before	After	Before	After	
Ⅰ	NA	NA	6.8	5.6	2.2	3.9	37	756	6400	960	12.8	5.2	11.3	3.1	88.2	59.2	93.7	23.7	7.65	6.92	37.8	38.5	Inadequate
Ⅱ	NA	NA	3.6	3.6	1.7	1.1	380	8	190	50	16.3	12.8	14.5	10.6	88.9	82.3	18.1	2.2	1.27	0.49	37.9	36.5	Improvement
Ⅲ	P	N	1.4	3.9	4.3	0.7	7600	170	9600	270	22.7	9.4	20.5	7.5	90.3	80.2	24.3	11.4	0.63	0.52	38.3	36.6	Improvement
Ⅳ	NA	NA	2.3	3.6	1.7	2.1	110	30	90	70	9.07	8.61	7.2	6.67	79.8	77.4	9.6	4.5	0.49	0.14	37.7	36.8	Improvement
Ⅴ	P	N	4.5	3.5	0.6	0.9	160	0	1920	0	12.2	6.7	10.4	4.4	85.1	65.9	51.7	7.2	44.87	4.20	37.4	37	Improvement
Ⅵ	P	NA	4	5.9	1	0.8	261	0	290	20	6.6	6.7	4.1	4.8	61.6	71.4	99.0	5.4	0.36	0.25	37.2	36.6	Improvement
Ⅶ	NA	NA	3.4	2.8	1.6	1.4	1400	220	640	20	15.9	8	12.2	4.9	77.1	61.4	32.4	2.5	0.63	0.34	37.4	36.5	Improvement
Ⅷ	NA	NA	0.7	2.1	2.1	0.9	1320	230	590	87	8.4	6.6	7.8	5.5	93.3	83.1	0.7	0.4	1.50	0.32	38	36.8	Improvement
Ⅸ	P	N	0.4	2.4	2.5	1.3	1600	20	7300	0	11.9	9.21	9.5	6.54	79.5	71	266.9	41.3	2.52	1.21	38.1	37	Improvement
Ⅹ	P	N	0.8	3.9	3.9	0.5	2640	1	70	0	7.8	5.3	6.5	4.3	83.8	81.8	84.4	29.6	1.30	0.41	37.2	36.4	Improvement

^a^ The reference ranges for these parameters: CSF, glucose, 2.2–3.9 mmol/L; CSF, protein, < 0.5 g/L; CSF WBC, count, 0–10 × 106/L; CSF RBC count; CSF, red blood cell count, 0 × 106/L; Blood WBC count, blood white blood cell count, 3.5–9.5 × 109/L; NEU#, absolute value of neutrophil, 1.8–6.3 × 109/L; NEU%, neutrophil percentage, 40%–75%; CRP, C-reactive protein, <5.0 mg/L; PCT, procalcitonin, 0.00–0.51 ng/mL; P, positive; N, negative; NA, the CSF, culture were not performed.

^b^ Before, 1 day before antimicrobial administration; After antibiotic treatment, he final day of antimicrobial therapy.

## 4 Discussion

We presented CSF meropenem, vancomycin and tigecycline monitoring in ten patients with bacterial CNSI following neurosurgery. The use of these three antibiotics for the treatment of CNSI was all based on current evidence and expert consensus (Tunkel et al., 2017). The time to post-operative CNSI was 3 to 18 days, similar to the published study (Chang et al., 2018).

### 4.1 Meropenem CSF pharmacokinetics and the impact of BBB permeability

Seven patients used meropenem alone or in combination. Amongst them, the CSF concentration-time profiles for Patients Ⅰ, Ⅱ, and Ⅹ revealed an estimated T_max_ of 2–3 h. Given that the current breakpoint for common Gram-negative bacteria causing CNSIs is 2 μg/mL, the percentage of time that meropenem concentrations are higher than the MIC (%T > MIC) for these three patients was 40.1%, 0%, and 64.4%, respectively (Alshaer et al., 2022). Age was also comparable between the three, so we found that an intravenous infusion of 2 g of meropenem every 8 h (Patient Ⅹ) was shown to provide benefits over a 1 g intravenous infusion regimen (Patient Ⅰ and Ⅱ) for treating CNSIs following neurosurgery. Even though the meropenem regimen was identical for Patients Ⅰ and Ⅱ, Patient Ⅰ (3.52 mg/mL) achieved a higher CSF concentration than Patient Ⅱ (2.01 mg/mL). Due to the lack of plasma collection for drug concentration measurements in these two patients, direct quantitative assessment of BBB permeability was not available. Therefore, this study compared CSF inflammatory biomarkers as reference indicators (Iguchi et al., 2020; Supplementary Table S2). The QAlb, a key metric of BBB integrity (Desestret et al., 2015), was higher in Patient I (0.079) than in Patient II (0.055), indicating more severe BBB disruption and higher permeability in Patient I. Furthermore, the CSF-to-serum glucose ratio was below 0.5 in Patient II, consistent with bacterial infection. In contrast, Patient I had a ratio exceeding 0.7, likely indicating stress-induced hyperglycemia from their traumatic injury and prolonged surgery. These same factors also contribute to direct BBB damage (Cash and Theus, 2020).

In total, six CSF C_trough_s of meropenem were monitored in Patients Ⅵ, Ⅶ and Ⅷ. The mean CSF C_trough_ was 1.24 ± 0.52 μg/mL (between 0.65 and 2.18 μg/mL). At post-surgery Days 8, 15, and 18, CSF meropenem levels in Patient Ⅷ were 0.65, 0.92, and 1.08 μg/mL, respectively. CSF vancomycin concentrations in the same individual also showed a significant increase from post-surgery Day 8 to Day 18 of 2.6 and 3.3 μg/mL. It was generally understood that with a longer period post-surgery, BBB disruption improved, and antibiotic CSF concentrations decreased (Fong et al., 2024). However, in Patient VIII with subarachnoid hemorrhage, CSF levels of meropenem and vancomycin increased over time post-surgery. This may be attributed to intracranial edema induced by both hemorrhage and craniotomy, which initially diluted antibiotic concentrations due to excess CSF volume. A comparable phenomenon has also been observed with antiepileptic drugs (Marchi et al., 2009). Cerebral edema is a major determinant of drug distribution in the brain, accompanied by a leakage phenomenon. As edema gradually resolved, CSF became more concentrated, leading to elevated drug levels. This trend contrasts with expectations from BBB recovery alone, highlighting the importance of considering edema dynamics in assessing antibiotic penetration after craniotomy. Notably, despite a higher plasma C_trough_, Patient VIII had a lower CSF C_trough_ than Patient VI. This distribution indicates greater BBB permeability in Patient VI (56.7%) than in Patient VIII (34.3%), which is consistent with the impact of cerebral edema in Patient VIII as previously described (Qin et al., 2015; Li et al., 2018). Both patients had similar liver and kidney function (Supplementary Table S1). Thus, the interpatient variability in drug exposure appears driven by differences in BBB integrity (e.g., edema, inflammation). One study appears to be inconsistent with our results, a meropenem C_CSF_/C_plasma_ ranging from 3% to 16% under mild or no meningeal inflammation (Blassmann et al., 2016). However, BBB penetration is closely associated with the degree of inflammation, and the AUC_CSF_/AUCplasma can reach up to 39% in severe cases (Nau et al., 2010). Therefore, the increased penetration observed in Patient VI and Patient VIII may be attributed to inflammation-compromised CSF outflow resistance, leading to drug accumulation and reduced clearance within the central nervous system (Nau et al., 2010).

### 4.2 Vancomycin penetration and the impact of administration route on CSF exposure

Vancomycin was used in six of ten patients, and ten CSF samples were collected. Four of these patients (Patients Ⅲ, Ⅳ, Ⅶ, and Ⅷ) received the vancomycin A regimen, and the mean CSF C_trough_ of them was 2.22 ± 0.87 μg/mL. These ranged in concentrations from 0.7 to 3.3 μg/mL. These results were similar to those reported in other studies, which revealed a CSF C_trough_ of 1.90 ± 1.29 μg/mL (range 0.42 to 4.40 μg/mL) for vancomycin patients receiving the same regimen (Cai et al., 2019). Vancomycin exhibited a BBB permeability of about 39.8% in patient Ⅷ, but this value was considerably higher than previous results obtained by Tiede, who demonstrated the BBB permeability was only 7% (Fan et al., 2022). This could be due to BBB disruption and inter-individual variation.

Studies have indicated that combined intrathecal and intravenous administration of vancomycin enhances treatment efficacy for CNSI without increasing the dosage (Ng et al., 2014; Remeš et al., 2013). In our study, Patient IX exhibited lower vancomycin trough concentrations than Patient VI in both CSF and plasma, despite both receiving the combined regimen. This discrepancy may be attributed to differences in renal clearance and administration sites. Patient IX had a slightly elevated eGFR (Supplementary Table S1), suggesting enhanced drug elimination. Furthermore, vancomycin was administered via the lateral ventricle in Patient VI and the lumbar spine in Patient IX. According to cerebrospinal fluid dynamics (Massaro et al., 2018), the lateral ventricle exhibits higher CSF flow and volume than the lumbar region, favoring drug distribution and potentially contributing to the higher concentration in Patient VI. The considerable interindividual variability in vancomycin penetration has also been documented in meningitis patients (Mader et al., 2018), underscoring the importance of therapeutic drug monitoring in CSF to optimize efficacy. Additionally, four patients treated with intravenous vancomycin alone exhibited trough concentrations higher than those in Patient IX but lower than in Patient VI, indicating that even intrathecal administration may not achieve adequate CSF concentrations in some patients, a finding that merits clinical attention.

### 4.3 Tigecycline penetration into CSF: inflammation and BBB disruption as potential facilitators

Tigecycline, a glycylcycline antibiotic, exhibits broad-spectrum activity against multidrug-resistant (MDR) and extensively drug-resistant (XDR) bacteria, particularly A. baumannii and *Klebsiella pneumoniae* (Yaghoubi et al., 2022; Townsend et al., 2011). Although ubiquitously distributed in different tissues, tigecycline exhibited little permeability at the BBB to achieve effective CSF concentrations. For patients who underwent neurosurgery, various invasive procedures (e.g., durotomy, hemicraniectomy) disturbed the BBB to some extent. In these disease states, whether tigecycline was able to partially cross the BBB was worthy of intensive investigation. The administration of tigecycline via the intrathecal route was successful for patients with CNSI, but there were no unified criteria for dosage and safety (Du et al., 2022). There have been relatively few studies on the tigecycline levels in CSF after intravenous injection, especially in CNSI or neurosurgery. The variability in CSF pharmacokinetic parameters, including concentration, AUC, and T_1/2_, is influenced by multiple factors. Since Patients V and X received identical tigecycline doses and administration, the observed differences likely stem from patient- and disease-related variables. Patient X, known after traumatic brain injury, may have caused more severe physical disruption of the BBB than Patient V. Elevated CSF-to-serum glucose ratio and CSF protein levels (Supplementary Table S2) suggest stronger inflammation in Patient X, which may increase BBB permeability (Klotz et al., 2016) and explain the higher tigecycline AUC and concentration in CSF. Elevated inflammatory factors also exacerbate the matrix effect in CSF (Cho et al., 2019). Additionally, which may account for the occurrence of several tigecycline concentrations that were above the LOD but below the LOQ. The presence of high liver enzyme parameters in Patient X, absent in Patient V, may explain the difference in drug half-life between the two patients. Different T_1/2_ might be closely associated with the various degrees of liver impairment in Patient Ⅹ and Patient Ⅴ. Upon reaching a therapeutic effect, both patients were CSF culture negative and experienced improvement in infection-related indices and body temperature. The intravenous infusion of tigecycline might exert therapeutic effects for patients with CNSI following neurosurgery.

### 4.4 Limitations of the study

Certainly, there are several limitations to the current study. All three antibiotics used were of special grade according to the Antibiotic Classification Management Lists in China, thus our sample size was small and constrained. Although the results suggest differences in the study, the observed associations have not been confirmed through rigorous statistical testing owing to limitations imposed by the small sample size and should therefore be interpreted only as a hypothesis awaiting validation. CSF and plasma samples were not collected simultaneously from the included ten patients. Analyses were not conducted by surgery type. The patients included did not all have positive CSF cultures before anti-infective treatment. Further studies in a larger, more diverse cohort are needed to confirm these findings.

## 5 Conclusion

The CSF concentration and PK/PD parameters of meropenem, vancomycin, and tigecycline in patients with CNSI following neurosurgery featured large inter-individual variation. Different drug regimens can partially improved the outcomes of such patients, but monitoring of potential adverse reactions is required.

## Data Availability

The original contributions presented in the study are included in the article/Supplementary Material, further inquiries can be directed to the corresponding authors.
