# Supplementary material for: Study on cerebrospinal fluid meropenem, vancomycin and tigecycline monitoring in patients with central nervous system infection following neurosurgery under different drug regimens
Source: Front Pharmacol. 2025 Sep 30;16:1666168. doi: 10.3389/fphar.2025.1666168 (PMC12518061; doi:10.3389/fphar.2025.1666168)
Supplement: Supplementary file 1 [file Supplementaryfile1.docx]

| **Supplementary Table S1 Results for renal clearance and liver function parameters^a^** | | | | | | | | | |
| --- | --- | --- | --- | --- | --- | --- | --- | --- | --- |
| **Patient ID** | **Gender** | **Age,  years** | **Time Point,  days** | **Creatinine,  µmol/L** | **eGFR,  mL/min/1.73m²** | **TBil, μmol/L** | **Albumin, g/L** | **PT, h** | **Child-Pugh Score** |
| Ⅰ | F | 65 | 0 | 45 | **103.57** | 10.1 | 27.9 | 13.7 | **B** |
| Ⅱ | M | 67 | -2 | 69 | **97.72** | 4.3 | 30.7 | 15.0 | **B** |
| Ⅲ | M | 47 | -10 | 124 | **62.24** | 16.2 | 38.8 | 14.2 | **A** |
| Ⅳ | F | 71 | -3 | 57 | **94.25** | 9.6 | 41.5 | 14.0 | **A** |
| Ⅴ | F | 43 | -1 | 32 | **128.93** | 14.8 | 29.5 | 13.9 | **A** |
| Ⅵ | F | 62 | 0 | 39 | **109.23** | 13.1 | 36.5 | 14.9 | **A** |
| Ⅶ | M | 54 | -5 | 60 | **110.52** | 9.0 | 31.2 | 13.6 | **A** |
| Ⅷ | F | 56 | -1 | 33 | **118.04** | 7.8 | 33.3 | 11.7 | **A** |
| Ⅸ | M | 50 | -1 | 47 | **121.98** | 11.0 | 32.4 | 15.6 | **B** |
| Ⅹ | M | 68 | -2 | 49 | **107.69** | 14.7 | 31.2 | 15.9 | **A** |
| **^a^Time Point, days of the most recent serum creatinine sample collected before drug administration; Renal clearance is assessed using eGFR. eGFR, estimated glomerular filtration rate, eGFR=142×(Creatinine/κ)^α^×0.9938^Age^, sex-specific coefficients: κ (0.9 for males and 0.7 for females) and α (-0.302 for males and -0.241 for females);  TBil, Total Bilirubin; PT; Prothrombin Time;Child-Pugh score was used to comprehensively assess liver function severity based on five clinical parameters: total bilirubin, serum albumin, prothrombin time , ascites, and hepatic encephalopathy. Scores are classified into three grades (A, B, and C) to evaluate liver function.** | | | | | | | | | |

| **Supplementary Table S2 The meningeal  inflammatory state of the patient** | | | | | |
| --- | --- | --- | --- | --- | --- |
| **Patient ID** | **Age,  years** | **CSF Protein,  g/L** | **CSF WBC Count, × 10^6^/L** | **QAlb^a^** | **CSF-to-serum  Glucose Ratio** |
| Ⅰ | 65 | 2.2 | 37 | 0.079 | 0.979 |
| Ⅱ | 67 | 1.7 | 380 | 0.055 | 0.434 |
| Ⅲ | 47 | 4.3 | 7600 | 0.111 | 0.307 |
| Ⅳ | 71 | 1.7 | 110 | 0.041 | 0.387 |
| Ⅴ | 43 | 0.6 | 160 | 0.02 | 0.669 |
| Ⅵ | 62 | 1 | 261 | 0.031 | 0.224 |
| Ⅶ | 54 | 1.6 | 1400 | 0.046 | 0.782 |
| Ⅷ | 56 | 2.1 | 1320 | 0.063 | 0.135 |
| Ⅸ | 50 | 2.5 | 1600 | 0.071 | 0.05 |
| Ⅹ | 68 | 3.9 | 2640 | 0.125 | 0.102 |
| **^a^QAlb, age-dependent albumin quotient. ^b^CSF and serum samples collected on the same date were collected simultaneously.** | | | | | |


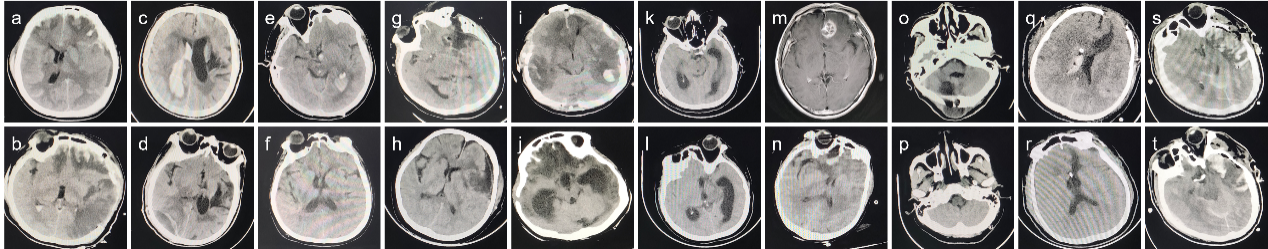
**Supplementary Figure 1 Radiological images of ten patients** Panels show representative images from Patients I through X before and after treatment: (a, b) Patient I; (c, d) Patient II; … continuing sequentially to Patient X. All ten patients showed varying degrees of improvement after surgery. Notably, Patients II through X exhibited significant reduction in lesion size and marked overall intracranial improvement.

| **Supplementary Table S3 The supplementary pharmacokinetic parameters of meropenem and tigecycline in CSF^a^** | | | | | |
| --- | --- | --- | --- | --- | --- |
| **Patient ID** | **Drug** | **MRT_0-∞_, h** | **VRT_0-∞_, h^2^** | **Vd, L** | **CL, L/h** |
| Ⅰ | Meropenem | 3.32 | 6.33 | 177.464 | 71.641 |
| Ⅱ |  | 8.17 | 50.99 | 389.052 | 55.065 |
| Ⅹ |  | 4.53 | 9.39 | 269.412 | 94.388 |
| Ⅴ | Tigecycline | 11.13 | 55.10 | 1.282 | 0.169 |
| Ⅹ |  | 12.62 | 138.68 | 0.738 | 0.062 |
| Mean ± SD |  | 5.34 ± 2.53 | 22.24 ± 24.95 | 278.64 ± 106.10 | 73.7 ± 19.74 |
| **^a^MRT_0-∞_, mean residence time from zero to infinity; VRT_0-∞_, variance of residence time from zero to infinity; Vd, volume of distribution; CL, aclearance.** | | | | | |

| **Supplementary Table S4 Antibiotic Susceptibility Test Results of CSF Cultures*** | | | | | | | | | | | | | | | |
| --- | --- | --- | --- | --- | --- | --- | --- | --- | --- | --- | --- | --- | --- | --- | --- |
| **Patient ID** | **Ⅲ** | | | **Ⅴ** | | | | | | | **Ⅵ** | **Ⅸ** | **Ⅹ** | | |
| **CSF Cultures** | *Sep* | | | *STM* | | *Sep* | | | *Efa* | | *P. larvae^e^* | *B. altitudinis^e^* | *A. baumannii* | | |
| **Susceptibility Test** | KB^b^,  mm | MIC^c^, μg/mL | Drug Susceptibility^d^ | KB,  mm | Drug Susceptibility | KB,  mm | MIC,  μg/mL | Drug Susceptibility | KB,  mm | Drug Susceptibility | KB,  mm | KB,  mm | KB,  mm | MIC,  μg/mL | Drug Susceptibility |
| **Penicillin** | 29 |  | S |  |  | 13 |  | R | 20 | S | 6 | 32 |  |  |  |
| **Levofloxacin** | 27 |  | S | 27 | S | 16 |  | I | 20 | S | 6 | 26 |  | 0.5 | S |
| **Moxifloxacin** | 28 |  | S |  |  | 21 |  | I |  |  | 6 | 26 |  |  |  |
| **Oxacillin** |  | 0.25 | S |  |  |  | 4 | R |  |  | 6 | 17 |  |  |  |
| **Gentamicin** | 26 |  | S |  |  | 25 |  | S | 17 | S | 6 | 21 |  |  |  |
| **Erythromycin** | 6 |  | R |  |  | 25 |  | S |  |  | 6 | 26 |  |  |  |
| **Clindamycin** | 6 |  | R |  |  | 23 |  | S |  |  | 6 | 14 |  |  |  |
| **Achromycin** | 25 |  | S |  |  | 21 |  | S |  |  | 6 | 23 |  |  |  |
| **Vancomycin** |  | 0.5 | S |  |  |  | 0.5 | S | 18 | S | 24 | 18 |  |  |  |
| **Linezolid** | 26 |  | S |  |  | 30 |  | S | 24 | S | 31 | 27 |  |  |  |
| **Teicoplanin** | 15 |  | S |  |  | 15 |  | S | 16 | S | 23 | 16 |  |  |  |
| **SMZ/TMP^a^** | 27 |  | S | 26 | S | 6 |  | R |  |  | 6 | 29 |  | 160 | R |
| **Rifampicin** | 31 |  | S |  |  | 30 |  | S | 12 | R | 13 | 18 |  |  |  |
| **Minocycline** |  |  |  | 28 | S |  |  |  | 23 | S |  |  |  | ≤1 | S |
| **Ampicillin** |  |  |  |  |  |  |  |  | 20 | S |  |  |  |  |  |
| **Piperacillin/Trizabalat** |  |  |  |  |  |  |  |  |  |  |  |  |  | ≥128 | R |
| **Ceftazidime** |  |  |  |  |  |  |  |  |  |  |  |  |  | ≥64 | R |
| **Cepharosporin/sulbactam** |  |  |  |  |  |  |  |  |  |  |  |  |  | 32 | I |
| **Cefepime** |  |  |  |  |  |  |  |  |  |  |  |  |  | 16 | I |
| **Imipenem** |  |  |  |  |  |  |  |  |  |  |  |  |  | 2 | S |
| **Meropenem** |  |  |  |  |  |  |  |  |  |  |  |  |  | 2 | S |
| **Nebcin** |  |  |  |  |  |  |  |  |  |  |  |  |  | ≥16 | R |
| **Ciprofloxacin** |  |  |  |  |  |  |  |  |  |  |  |  |  | 0.5 | S |
| **Deoxytetracycline** |  |  |  |  |  |  |  |  |  |  |  |  |  | ≤ 0.5 | S |
| **Tigecycline** |  |  |  |  |  |  |  |  |  |  |  |  |  | 1.0 | S |
| **Colistin** |  |  |  |  |  |  |  |  |  |  |  |  |  | ≤0.5 | S |
| **Piperacillin** |  |  |  |  |  |  |  |  |  |  |  |  | 6 |  | R |
| **Amoxicillin/sulbactam** |  |  |  |  |  |  |  |  |  |  |  |  | 10 |  | R |
| **Amikacin** |  |  |  |  |  |  |  |  |  |  |  |  | 6 |  | R |
| **Cefatriaxone** |  |  |  |  |  |  |  |  |  |  |  |  | 6 |  | R |
| **^a^SMZ/TMP， Paediatric Compound Sulfamethoxazole Tablets； ^b^K-B, Kirby-Bauer; ^c^MIC， Minimal inhibitory concentration; ^d^Drug susceptibility was interpreted and categorized according to the Clinical and Laboratory Standards Institute (CLSI) M100 2020 guidelines into three categories: S, susceptible; I, intermediate ;R, resistant; ^e^According to CLSI, there are no established interpretive criteria for this bacterium *The absence of data indicates that testing is not deemed necessary for this organism-drug combination.** | | | | | | | | | | | | | | | |

| **Supplementary Table S5 Methodological investigation  for each assay^a^** | | | |
| --- | --- | --- | --- |
| **Antibacterial** | **LOD** | **LOQ** | |
|  |  | **CSF** | **Plasma** |
| Meropenem, μg/mL | 0.10 | 0.25 | 1.00 |
| Vancomycin, μg/mL | 0 | 0 | 0 |
| Tigecycline, ng/mL | 15.000 | 49.305 | 75.226 |
| **^a^LOD, limit of detection； LQD, limit of quantitation.** | | | |

| **Supplementary Table S6 Accuracy and precision of drug determination assays^a^** | | | | | | | | |
| --- | --- | --- | --- | --- | --- | --- | --- | --- |
| **Drug** | **Assay** | **Sample  Type** | **Low/Medium/High  Concentrations  (μg/mL)** | **Accuracy (Relative Recovery,  Mean (%) ± SD, *n* =5)** | | | **Precision (RSD^a^, %)** | |
|  |  |  |  | **Low** | **Medium** | **High** | **Intra-Day Precision (n =5)** | **Inter-Day Precision (n = 5)** |
| Meropenem | HPLC | CSF | 0.75/2.5/20 | 98.02±4.47 | 106.17±2.12 | 100.77±0.75 | 2.6 | 5.8 |
| Vancomycin | Enzyme Immunoassay |  | 0/15/50 | 100.84±0.79 | 101.20±1.41 | 99.57±2.35 | 1.9 | 3.9 |
| Tigecycline | Two-dimensional LC |  | 147.92/730.44/2536.24 | 96.39±5.19 | 102.08±3.55 | 99.47±4.16 | 0.7 | 4.9 |
| Meropenem | HPLC | Plasma | 3/10/80 | 102.37±7.97 | 102.63±6.10 | 98.27±6.79 | 6.5 | 8.1 |
| Vancomycin | Enzyme Immunoassay |  | 0/30/75 | 100.06±2.13 | 95.68±3.19 | 97.69±5.42 | 1.5 | 5.4 |
| Tigecycline | Two-dimensional LC |  | 225.66/1206.00/3569.12 | 98.16±2.92 | 101.99±4.59 | 100.74±5.56 | 5.2 | 5.5 |
| **^a^Guidelines of the FDA for Bioanalytical Method Validation 2018 were followed for method validation； ^b^RSD (Relative Standard Deviation) and CV (Coefficient of Variation) have the same meaning.** | | | | | | | | |

As summarized in Supplementary Table S6, all assays demonstrated acceptable accuracy and precision. The mean accuracy (relative recovery) ranged from 95.68% to 106.17%, and both intra-day and inter-day precision (RSD) were within 8.1% across all quality control levels (low, medium, and high) for each analyte and matrix. Sensitivity confirmed through multiple validation batches. Both precision and accuracy at the LOQ level met the recommended criteria, with all CV values below 20% and percent bias within ±20%.
